# Do GNAQ and GNA11 Differentially Affect Inflammation and HLA Expression in Uveal Melanoma?

**DOI:** 10.3390/cancers11081127

**Published:** 2019-08-07

**Authors:** Christiaan van Weeghel, Annemijn P. A. Wierenga, Mieke Versluis, Thorbald van Hall, Pieter A. van der Velden, Wilma G. M. Kroes, Ulrich Pfeffer, Gregorius P. M. Luyten, Martine J. Jager

**Affiliations:** 1 Department of Ophthalmology, Leiden University Medical Center, Albinusdreef 2, 2333 ZA Leiden, The Netherlands; 2 Department of Clinical Oncology, LUMC, 2333 ZA Leiden, The Netherlands; 3 Department of Clinical Genetics, LUMC, 2333 ZA Leiden, The Netherlands; 4 Tumor Epigenetics, IRCCS Ospedale Policlinico San Martino, 16132 Genova, Italy

**Keywords:** uveal melanoma, mutations, chromosome aberrations, mRNA expression, survival

## Abstract

Inflammation, characterized by high numbers of infiltrating leukocytes and a high HLA Class I expression, is associated with a bad prognosis in uveal melanoma (UM). We wondered whether mutations in *GNA11* or *GNAQ* differentially affect inflammation and HLA expression, and thereby progression of the disease. We analyzed data of 59 primarily enucleated UM eyes. The type of *GNAQ*/*11* mutation was analyzed using dPCR; chromosome aberrations were determined by Fluorescence in Situ Hybridization (FISH), karyotyping, and single nucleotide polymorphism (SNP) analysis, and mRNA expression by Illumina PCR. Comparing tumors with a *GNAQ* mutation with those with a *GNA11* mutation yielded no significant differences in histopathological characteristics, infiltrate, or HLA expression. When comparing the Q209L mutations with Q209P mutations in tumors with monosomy of chromosome 3, a higher mitotic count was found in the Q209P/M3 tumors (*p* = 0.007). The Kaplan-Meier (KM) curves between the patients of the different groups were not significantly different. We conclude that the type (Q209P/Q209L) or location of the mutation (*GNA11*/*GNAQ*) do not have a significant effect on the immunological characteristics of the tumors, such as infiltrate and HLA Class I expression. Chromosome 3 status was the main determinant in explaining the difference in infiltrate and HLA expression.

## 1. Introduction

Uveal melanoma (UM) is a form of cancer that occurs mostly in people aged 60 years and older and has a prevalence of around 5.0 per million people within The Netherlands [1]. Unfortunately, between 30% and 50% of the people affected by UM eventually die due to metastases [2]. UM forms in the uvea, which is made up of the iris, the ciliary body, and the choroid. At this moment in time, the most common treatments of UM are enucleation, plaque irradiation, and proton therapy.

The primary *GNAQ*/*GNA11* mutation in UM occurs in the melanocyte and is already present in choroidal nevi [3,4,5]. During malignant progression, this genetic modification is usually followed by gain of chromosome 6q or gain of 8q, as well as a mutation in the *BAP1*, *EIF1AX*, or *SF3B1* gene [6,7,8], and/or a complete loss of one chromosome 3 (monosomy 3 (M3)). M3 loss and mutations in *BAP1* often co-occur and are associated with a bad prognosis [9,10,11,12,13,14]. Chromosome 6q gain is associated with a better prognosis and a lower chance of developing metastatic disease [15]. Gain of 8q is associated with metastases but often occurs together with monosomy 3. The specific sequence of events with regard to BAP1 and chromosome 3 is not yet clear, while it is likely that gain of 8q often develops prior to loss of chromosome 3 [16,17,18].

It is thought that UM cells have a survival advantage because of the tumor’s location in the immune-privileged eye: tumor cells would not primarily induce a systemic anti-tumor immune response but might even inhibit the development of anti-tumor immunity [19]. However, especially prognostically-bad UM often display an inflammatory phenotype which is characterized by the presence of immune cells such as T lymphocytes, macrophages, and an increased HLA expression [20]. The presence of such an inflammatory phenotype is associated with a bad prognosis [13,21,22,23].

HLA Class I (A–C and E–G) molecules present tumor proteins to T cells, while HLA Class II molecules (DM, DO, DP, DQ and DR) present proteins that originate from outside the cell to T cells. If the T cells recognize a presented protein that does not belong to the normal set of proteins, an immune reaction can develop. Infiltrating T cells in UM do not prevent the tumor from developing because the infiltrate is seen as a factor associated with a poor prognosis [23,24]. From previous research it is known that HLA-A and HLA-B expression is higher in patients with an M3 status [23]. Natural killer (NK) cells cannot attack the tumor due to the high amount of HLA expressed on UM cells, which inhibits their function, and due to the presence of high levels of macrophage migration inhibitory factor (MIF) and TGF-β in the aqueous humor, which inhibit NK cell function [19]. HLA-G can help cancer cells escape the immune system and can also inhibit NK cell-mediated lysis [25]. Due to the lack of response of the immune system to the tumor, the tumor will continue to grow [19,26]. However, research from our lab has shown a low expression of HLA-G [27]. From previous research it is known that HLA-A and HLA-B expression is higher in patients with an M3 status [23]. We have previously noticed that an increase in the number of macrophages in the disomy 3 (D3) group is related to the presence of an extra copy of chromosome 8q [28].

M3/*BAP1*-mutated tumors have distinct genomic signaling and immune profiles [13] and are often associated with high numbers of infiltrating leukocytes and macrophages. However, a great variability in infiltrate has been observed within the M3 and also in the D3 group. We wondered whether the expression of different types of HLA molecules in UM is similarly related to loss of chromosome 3 or might be related to the type of basic mutation (*GNAQ* or *GNA11* mutation or the type of mutation (Q209P/Q209L)). *GNAQ* and *GNA11* are genes encoding subunits of G-protein-coupled receptors (GPCRs), transmembrane receptors that serve as signal transducers operating from the cell membrane. They require glutamine in position 209 (Q209) to function properly. Without glutamine on Q209, the GPCR cannot hydrolyze Guanosine Triphosphate (GTP) to Guanosine Diphosphate (GDP) [4,29]. Q209 is a hotspot for missense mutations where the proteins translated from *GNAQ*/*GNA11* often have glutamine replaced by proline (Q209P mutation) or by leucine (Q209L mutation). We set out to determine whether the difference between a *GNAQ* or *GNA11* mutation or the type of mutation in these genes (Q209L versus Q209P) influenced the level of HLA expression and infiltrate in UM.

## 2. Results

### 2.1. mRNA and Patient Distribution

**Figure 1:**
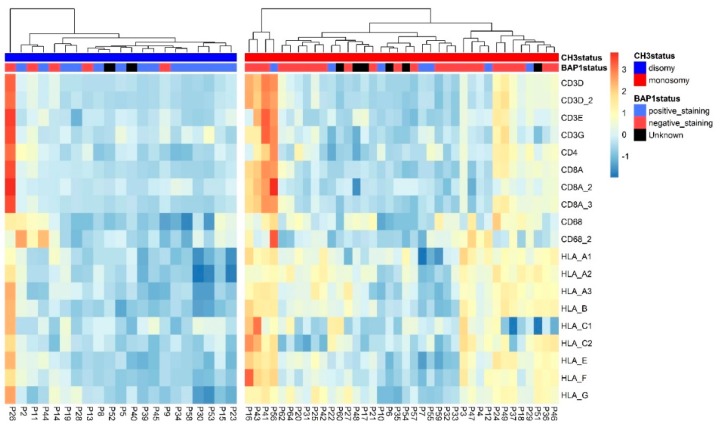
Two heatmaps showing the expression of several infiltrate and HLA Class I markers, normalized per marker over all 59 tumors. On the left is the heatmap of the disomy 3 (D3) tumors (*n* = 21) and on the right the heatmap of the monosomy 3 (M3) tumors (*n* = 38); tumors are clustered according to the amount of infiltrate and HLA Class I expression. Annotated are the chromosome 3 status (blue for D3 tumors, red for M3 tumors) and BAP1 staining. The presence of positive nuclear staining for BAP1 is indicated in blue (BAP1 is normal), while red indicates no BAP1 staining and black an unknown BAP1 status.

We first compared the expression of different HLA molecules with the tumor’s chromosome 3 status using mRNA expression data acquired from an Illumina chip: in Figure 1, it is shown that M3 tumors differ greatly from disomy 3 (D3) tumors with regard to inflammatory markers, with more of the M3 tumors showing high numbers of infiltrating leukocytes and a high HLA Class I expression. However, within each group, tumors differ significantly in infiltrate and HLA expression (Figure 1). For illustration, tumors were placed into a D3 and M3 cluster, and then into clusters through unsupervised clustering, based on the inflammatory and HLA markers within the D3 and M3 groups.

We set out to determine if mutations in *GNAQ* and *GNA11* are responsible for different degrees of inflammation within the D3 or M3 groups. The comparisons of clinical and histopathological information can be seen in Table 1 and Table 2: Table 1 contains the tumors with *GNAQ* or *GNA11* mutations, while Table 2 contains the data of tumors identified by Q209L and Q209P mutations. These data show that the clinical and histopathological characteristics did not differ significantly between mutations.

We subsequently set out to investigate whether the differences in levels of infiltrating leukocytes or in HLA expression were related to the presence of *GNAQ*/*GNA11* mutations (Table 3), or the type of mutation Q209L/Q209P (Table 4). No differences were observed in the expression of inflammatory markers such as HLA expression or levels of infiltrating leukocyte. However, significant differences in HLA expression and some infiltrate markers were seen between tumors with D3 or M3.

### 2.2. Survival

**Figure 2:**
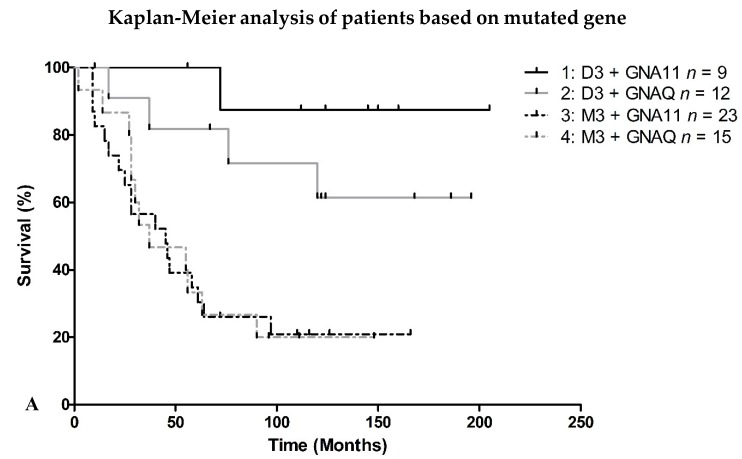

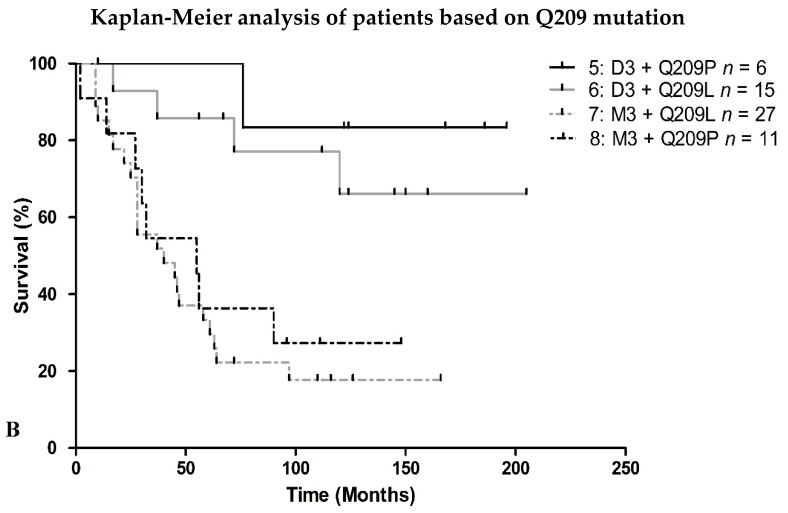
Survival of 59 UM patients, split depending on the location of the mutation (**A**) or type of mutation (**B**). *p*-values for the differences between the groups calculated with the log-rank function were (**A**): **1 versus** **2**: *p* = 0.47, **1 versus 3**: *p* < 0.001, **2 versus 4**: *p =* 0.030, **3 versus 4**: *p =* 0.90; (**B**): **5 versus 6**: *p =* 0.77, **6 versus 7**: *p* = 0.002, **5 versus 7**: *p* = 0.007, **7 versus 8**: *p* = 0.07.

Additionally, the survival of the different patient-groups was examined. A difference in survival could indicate a difference between the tumors that is not seen using these mRNA probes (Figure 2a,b). In Figure 2a,b there is no significant difference between the *GNA11*/*GNAQ* or Q209L/Q209P mutations. However, as expected, the data shows a significant association with M3 status.

### 2.3. HLA Expression in Relation to Chromosome 8q Status

**Figure 3:**
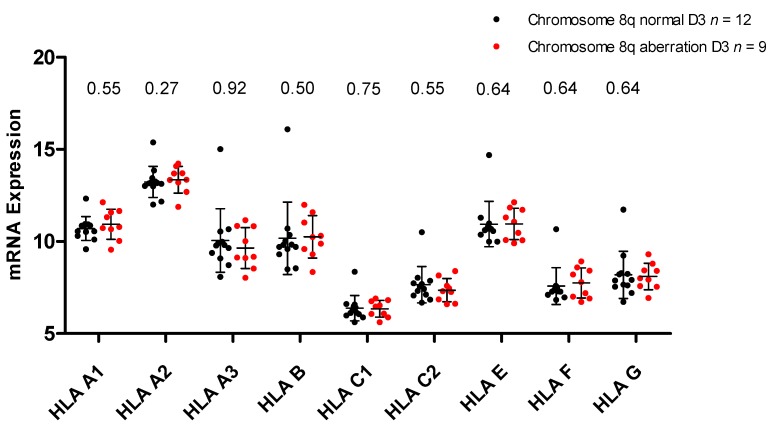
mRNA expression of all D3 tumors split on chromosome 8q status, shown for multiple probes (*p*-values shown were obtained using the Mann-Whitney U-test, comparing D3/chr8q normal (*n* = 12) with D3/Chr8q aberrant tumors (*n* = 9)).

Instead of a *GNAQ*/*11* mutation, a difference in chromosome 6 or 8q status might influence HLA expression. Gezgin has shown that the presence of macrophages is associated with 8q [28]. While almost all M3 tumors also have additional copies of 8q, there is variety between D3 tumors. However, there is no significant difference in HLA expression between D3 tumors with or without chromosome 8q aberrations (Figure 3).

## 3. Discussion

Despite the small group sizes for *GNA11* and *GNAQ* tumors it is quite clear that there is no significant difference in the expression patterns of HLA Class I and the presence of T cells or macrophages between tumors with either a *GNAQ* or *GNA11* mutation or a Q209P or Q209P mutation. Therefore, there is no evidence that the differences in inflammation found in groups with the same chromosome 3 status are caused by the different mutations on *GNAQ* or *GNA11* or the type of mutation Q209L or Q209P. The literature also suggests mutations on *GNAQ* do not affect the survival, and this was confirmed in this study, as no difference between *GNAQ*/*GNA11* was observed [30]. Due to Q209 being crucial for GTPase activity, GTP hydrolysis is abolished in both types of mutation in both genes [31]. As we did not find any difference, there is probably no difference in any pathway activation between the different mutations, or any different secondary effect of the sub-units formed from *GNA11* and *GNAQ*. If a specific mutation would have changed the protein into one that remained functional but with less affinity, the different mutations could have explained the difference in inflammation. We did not further study any relation with the Yes-Associated Protein (YAP) pathway as we did not find any differences related to the *GNAQ*/*11* mutations.

M3 and D3 tumors showed a significant difference in expression with regard to most T cell and macrophage probes. This was expected based on previous research [23,28,32]. However, we now show that the expression of all the different HLA Class I molecules is related to the tumor’s chromosome 3 status. However, both within the D3 group as well as within the M3 group, some tumors were observed to have more inflammation than others, indicating that there must also be other factors that have yet to be identified, besides the chromosome aberrations. 

**Figure 4:**
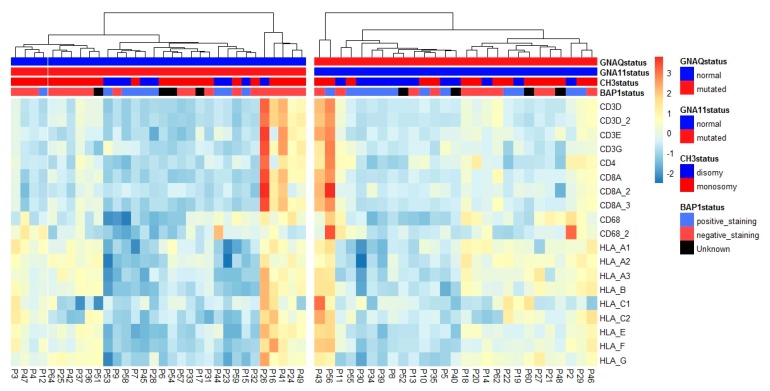
Heatmap of tumors split according to *GNAQ*/*GNA11* mutation. The heatmap shows the expression of several infiltrate and HLA Class I markers, normalized per marker over all 59 tumors. Annotated is the chromosome 3 status (blue for D3 tumors, red for M3 tumors). BAP1 staining is annotated below the chromosome 3 status. Positive staining for BAP1 is blue (BAP1 is normal), red is given for absent staining and black is given for unknown BAP1 status. Mutations on *GNAQ*/*GNA11* only include Q209L and Q209P mutations. GNAQ mutated: *n* = 27, GNA11 mutated: *n* = 32.

However, our data indicates that *GNAQ* and *GNA11* do not play a direct role in regulating inflammation. This is reflected in Figure 4. If the heatmap from Figure 1 is rearranged according to the *GNAQ*/*GNA11* mutation status, no obvious difference is seen between the *GNA11*-mutated tumors on the left and the *GNAQ*-mutated tumors on the right. Previous work on *GNAQ* and *GNA11* has shown that they activate the G protein signaling cascade, leading to the stimulation of Mitogen-Activated Protein (MAP) kinases, protein kinase B (Akt), protein kinase C (PKC), and Rho GTPase [33,34]. Blocking of PKC activity has been associated with downregulation of the nuclear factor kappa-light-chain-enhancer of activated B cells (NFKB) pathway, one of the leading regulators of HLA expression [35,36], while Rho GTPase has been associated with HIF1a upregulation, also leading to NFKB activity [37]. As we saw differences in inflammation between tumors with the same chromosome 3 status, we considered the option that the two types of mutations might differentially affect this inflammation. Although there was no difference, it is quite possible that both mutations similarly stimulate an inflammatory environment in melanocytes, such as nevi [5].

The heatmap in Figure 4 clearly shows that several groups with different levels of inflammation and HLA expression exist in the different *GNAQ* or *GNA11* mutated tumors. These differences are not all related to the chromosome 3 status. However, it is yet unknown what causes the cause of the inflammation within the tumors with the same chromosome 3 status.

Finding the cause of the relation between M3 and inflammation may help us to better understand how inflammation is involved in spreading metastasis.

Any differences found between the groups could be caused by other rarer secondary mutations but are not caused by a *GNAQ/GNA11* mutation. We do not find a difference in survival between the tumors with different *GNAQ/GNA11* mutations, while a clear difference can be observed between patients with a different chromosome 3 status. How the loss of chromosome 3/BAP1 leads to an inflammatory phenotype will be the subject of further studies.

## 4. Materials and Methods

### 4.1. Cases

A total of 64 tumor samples were collected from enucleated eyes at the Department of Ophthalmology at the Leiden University Medical Centre (LUMC) in Leiden, the Netherlands. All tumor samples were from patients who underwent primary enucleation for UM between 1999 and 2009. The collection of materials and research protocol were compliant with the tenets of the declaration of Helsinki (World Medical Association of Declaration 2013; ethical principles for medical research involving human subjects). Tumor material was handled in accordance with the Dutch National Ethical Guidelines (‘Code for Proper Secondary Use of Human Tissue”).

The pathological data was obtained from patient charts. The tumors were examined by a pathologist specializing in ocular-oncology. 

The Medisch Ethische Toetsingscommissie (METC) gave approval on 19 October 2016 with the code G16.076/NV/gk. 

### 4.2. Chromosome Analysis

Following enucleation, small parts of the tumor were sent out for cell culturing [16]. These cells were cultured and those that were successfully cultured had karyotyping performed on them. Some of the tumors also had a FISH analysis performed on them.

Samples collected at the Leiden University Medical Center underwent DNA isolation using a QIAmp DNA Mini Kit (Qiagen, Venlo, the Netherlands) following the manufacturer’s guidelines.

An SNP analysis was performed on all 64 analyzed tumors. For the SNP assay, two microarray chips were used: the Affymetrix 250K_NSP-chip, which holds approximately 250,000 probes across the genome, and the Affymetrix Cytoscan HD chip, which holds approximately 750,000 probes across the genome. The copy number was determined using the “genotyping console (GTC)” (Affymetrix, Santa Clara, CA, USA). The “GTC Browser” (Affymetrix, Santa Clara, CA, USA) was used to visualize the data of the Affymetrix 250K_NSP. The Affymetrix Cytoscan HD chips were analyzed using the “Chromosome Analysis Suite” ‘ChaS’ (Affymetrix). The thresholds for chromosome aberrations were: <1.9 loss, 1.9–2.1 normal, >2.1 <3.1 gain, >3.1 amplification. In this research the loss, gain and amplifications were classified as chromosome aberrations.

In cases where karyotyping, FISH, or SNP disagreed, an abnormal situation was assumed (e.g., when karyotyping showed M3 status, even if FISH and SNP showed D3, an M3 status was assumed).

### 4.3. GNAQ and GNA11 Mutations

The presence of a mutation in either GNAQ/GNA11 was analyzed using hydrolysis probes in a duplex dPCR. Of each tumor sample 10 ng DNA was used in a 20 μL reaction volume. The protocol was performed as described before [16]. The reaction mixture consisted of 2× droplet PCR supermix (Bio-Rad Laboratories, Inc., Berkeley, CA, USA). 20× target probe (FAM), 20× wildtype probe (HEX). Proprietary probes and primers (Bio-Rad Laboratories, Inc.) were used. The following MiQE sequences:

**GNAQ Q209P** (ID: dHsaIS2501447 & ID: dHsaIS2501446):

TGCTATTTAAACTTGAACTCAAAGCCACCTATTTTGATACTATGTAAAAAATTATGTTGC

AAACTCACACCCTAAAACTTTTTCTTTAAAGAGGTATAACTGACATACTCAGAGAGAG

ATAAA

**GNAQ Q209L** (ID: dHsaCP2000052 & ID: dHsaCP2000051): 

AGTGTATCCATTTTCTTCTCTCTGACCTTTGGCCCCCTACATCGAC

CATTCTGCAAGGTTAACAATACTCATATTAATAACATATAAAGTAAA

ACTAAAAAGTCAACATAAATATAGCACTAC

**GNA11 209L** (ID: dHsaCP2000050 & ID: dHsaCP2000049):

CTTTCAGGATGGTGGATGTGGGGGGCCAGCGGTCGGAGCGGAGGAAGTGGATCCA

CTGCTTTGAGAACGTGACATCCATCATGTTTCTCGTCGCCCTCAGCGAATACGACCAA

GTCCTGGTGG

Using a QX100 droplet generator and DG8 cartridges (Bio-Rad Laboratories, Inc.), each 20 μL sample was converted to an emulsion of 20,000 droplets. The emulsion was then transferred to a 96-well PCR plate. The following end point PCR program was used to identify the mutations using a T100 Thermal cycler. 95 °C for 10 minutes, then 40 cycles of: 94 °C for 30 seconds, 55 °C for 1 min. Followed by 98 °C for 10 minutes and then it was kept at 4 °C until the results were read by a QX100 droplet reader (Bio-Rad Laboratories, Inc.). Digital PCR (dPCR) software (QuantaSoft, Berkeley, CA, USA) was used to read the results using fluorescence, and for analyzing the data. 

### 4.4. mRNA Analysis Status

mRNA status was obtained from fresh frozen tumor tissue. RNA for gene expression profiling was isolated with the RNeasy mini kit (Qiagen). HLA Class I expression was measured on an Illumina HT-12v4 chip (Illumina) following the manufacturer’s guidelines. The probes seen in Table 5 were used for this analysis. They had previously been compared with immunohistochemical data [24,28].

### 4.5. Statistical Analysis

To analyze the data several programs were used. Statistical package for social sciences (SPSS) (IBM Corp; released 2015; IBM SPSS Statistics for Windows, Version 23.0; Armonk, NY, IBM Corp) was used to analyze different groups and characteristics. All analyses were done with SPSS except those regarding the HLA expression. For differences between the groups related to chromosome 3 status, a Pearson’s χ^2^ test was used, and in case of more than two groups, a Fisher’s exact test. When numerical data was used such as the average age at time of enucleation, a two-tailed *t*-test was used and the *p*-value that did not assume equal variances was used.

MATLAB (MATLAB and Statistics Toolbox Release 2018a, The MathWorks, Inc., Natick, Massachusetts, USA) was used to analyze the differences in HLA expression between the different groups using the Wilcoxon Rank-sum test, and to compare survival curves. For the comparison between survival curves the following file was used Cardillo G. (2008). LogRank: Comparing survival curves of two groups using the log rank test http://www.mathworks.com/matlabcentral/fileexchange/22317) version 2.0.0.0

Kaplan-Meier curves were made using GraphPad Prism version 5.00 for Windows, GraphPad Software, La Jolla California USA, www.graphpad.com. 

The analyses were performed with data of 59 tumors of which the chromosome 3 status and the mutation of *GNAQ/GNA11* was known. M3 tumors were analyzed separately from D3 tumors. When separated on chromosome 3 status and GNAQ/11 mutations the following group sizes were created: D3 + GNA11 *n* = 9, D3 + GNAQ *n* = 12, M3 + GNA11 *n* = 23, M3 + GNAQ *n* = 15, D3 + Q209L *n* = 15, D3 + Q209P *n* = 6, M3 + Q209L *n* = 27, and M3 + Q209P *n* = 11.

## 5. Conclusions

The type and location of mutations on *GNAQ*/*GNA11* do not seem to affect the progression of UM. While these are driver mutations, there is no difference in mRNA expression of infiltrate markers and HLA Class I expression. Survival was also not significantly affected by these mutations. The chromosome 8q status did not explain HLA expression differences either. The main difference between inflamed and non-inflamed tumors is the chromosome 3 status. Chromosome 3 status also had a major influence on HLA expression. The *GNAQ/GNA11* mutations do not play a major role in distinguishing between tumors with and without an inflammatory phenotype. 

## Figures and Tables

**Table 1:** Comparison of 59 patients for whom HLA expression (mRNA), chromosome 3 status, and *GNAQ*/*GNA11* status was known. Significantly different values are represented in bold. The categorical groups were compared using a chi-squared test and numerical data were compared using an independent *t*-test.

Characteristics	D3 *GNA11* (*n* = 9)	D3 *GNAQ* (*n* = 12)	M3 *GNA11* (*n* = 23)	M3 *GNAQ* (*n* = 15)	Disomy 3 Tumors, *GNA11* versus *GNAQ* *p*-Value	Monosomy 3 Tumors, *GNA11* versus *GNAQ p*-Value	Monosomy 3 versus Disomy 3 *p*-Value
**Gender *n* (%)**					0.70 ^1^	0.85 ^1^	0.21 ^1^
Female	3 (33)	5 (42)	13 (57)	8 (53)			
Male	6 (66)	7 (58)	10 (44)	7 (47)			
**Survival *n* (%)**					0.11 ^1^	0.86 ^1^	0.001 ^1^
Alive	7 (78)	3 (25)	3 (13)	2 (13)			
Cause of Death:							
Melanoma-related	1 (11)	4 (33)	18 (78)	12 (80)			
Different cause	1 (11)	3 (25)	1 (4)	1 (7)			
Unknown	0 (0)	2 (17)	1 (4)	0 (0)			
**Affected eye *n* (%)**					0.70 ^1^	0.55 ^1^	0.28 ^1^
Right eye	3 (33)	5 (42)	13 (57)	7 (47)			
Left eye	6 (66)	7 (58)	10 (44)	8 (53)			
**Age at enucleation**					0.14 ^2^	0.65 ^2^	0.012 ^2^
*Mean*	47.24	59.11	63.95	65.91			
*±St. dev.*	±17.82	±16.75	±15.28	±10.67			
**Metastases *n* (%)**					0.24 ^1^	0.90 ^1^	<0.001 ^1^
No	8 (89)	8 (67)	5 (22)	3 (20)			
Yes	1 (11)	4 (33)	18 (78)	12 (80)			
**Cell type *n* (%)**					0.43 ^1^	0.78 ^1^	0.04 ^1^
Spindle	5 (56)	6 (50)	4 (17)	4 (26)			
Mixed	3 (33)	6 (50)	15 (65)	9 (60)			
Epithelioid	1 (11)	0 (0)	4 (17)	2 (13)			
**Basal diameter**					0.20 ^2^	0.08 ^2^	0.18 ^2^
*Mean (mm)*	12.00	13.42	13.13	15.67			
*±St. dev.*	±1.80	±3.09	±3.29	±4.62			
**Prominence**					0.81 ^2^	0.75 ^2^	0.30 ^2^
*Mean (mm)*	7.00	7.29	7.85	8.13			
*±St. dev.*	±2.18	±3.39	±2.89	±2.59			
**Mitotic count**					0.07 ^2^	0.13 ^2^	0.76 ^2^
*Mean ^3^*	9.00	4.58	5.52	9.29			
*±St. Dev.*	±5.83	±3.77	±3.40	±8.33			
**Ciliary body involvement *n* (%)**					0.72 ^1^	0.58 ^1^	0.009 ^1^
No	8 (89)	10 (83)	11 (48)	8 (57)			
Yes	1 (11)	2 (17)	12 (52)	6 (43)			
**Extra-ocular extension**					-	0.12 ^1^	0.67 ^1^
*<5 mm*	0 (0)	1 (50)	4 (75)	0 (0)			
*>5 mm*	0 (0)	1 (50)	1 (25)	1 (100)			
**Chr. 8q status *n* (%)**					0.10 ^1^	0.34 ^1^	<0.001 ^1^
Normal	7 (78)	5 (42)	4 (17)	1 (7)			
Aberrant	2 (22)	7 (58)	19 (83)	14 (93)			
**Chr. 6p status *n* (%)**					0.37 ^1^	0.57 ^1^	<0.001 ^1^
Normal	2 (22)	1 (8)	20 (87)	12 (80)			
Aberrant	7 (78)	11 (92)	3 (13)	3 (20)			

^1^ Pearson chi-squared test. ^2^ Two-tailed *t*-test (without equal variances). ^3^ mitosis/2 mm^2^. Evaluating the group sizes in a Fisher’s exact test yielded no significance either (*p*-value = 0.094). This means that the ratio of patients with M3/D3 is not significantly different between patients that have a mutation on a different gene.

**Table 2:** Comparison of 59 patients for whom HLA expression (mRNA), chromosome 3 status, and type of *GNAQ*/*GNA11* mutation was known. Significant values are shown in bold. The categorical groups were compared using a chi-squared test and numerical data was compared using an independent *t*-test.

Characteristics	D3 Q209L (*n* = 15)	D3 Q209P (*n* = 6)	M3 Q209L (*n* = 27)	M3 Q209P (*n* = 11)	Disomy 3 Tumors *Q209L* versus *Q209P* *p*-Value	Monosomy 3 Tumors *Q209L* versus *Q209P* *p*-Value	Monosomy 3 versus Disomy 3 *p*-Value
**Gender *n* (%)**					0.48 ^1^	0.96 ^1^	0.21 ^1^
Female	5 (33)	3 (50)	15 (56)	6 (55)			
Male	10 (66)	3 (50)	12 (44)	5 (45)			
**Age at enucleation**					0.92 ^2^	0.70 ^2^	0.15 ^2^
*Mean*	53.78	54.64	64.19	66.01			
*±St. dev.*	± 19.21	±15.39	±14.20	±12.23			
**Affected eye *n* (%)**					0.48 ^1^	0.39 ^1^	0.28 ^1^
Right eye	5 (33)	3 (50)	13 (48)	7 (64)			
Left eye	10 (66)	3 (50)	14 (52)	4 (36)			
**Survival *n* (%)**					0.88 ^1^	0.75 ^1^	0.001 ^1^
Alive	7 (47)	3 (50)	3 (11)	2 (18)			
Cause of Death:							
Melanoma-related	4 (27)	1 (17)	22 (81)	8 (73)			
Different cause	3 (20)	1 (17)	1 (4)	1 (9)			
Unknown	1 (7)	1 (17)	1 (4)	0 (0)			
**Metastases *n* (%)**					0.63 ^1^	0.55 ^1^	<0.001 ^1^
No	11 (73)	5 (83)	5 (19)	3 (27)			
Yes	4 (27)	1 (17)	22 (81)	8 (73)			
**Basal diameter**					0.34 ^2^	0.27 ^2^	0.30 ^2^
*Mean (mm)*	13.2	11.83	13.56	15.55			
*±St. dev.*	±2.6	±2.9	±3.3	±5.3			
**Prominence**					0.67 ^2^	0.56 ^2^	0.86 ^2^
*Mean (mm)*	6.97	7.67	7.80	8.36			
*±St. dev.*	±2.7	±3.5	±2.8	±2.7			
**Mitotic count**					0.29 ^2^	0.22 ^2^	0.96 ^2^
*Mean ^3^*	7.20	4.67	5.81	9.64			
*±St. dev.*	±5.38	±4.41	±3.37	±9.39			
**Ciliary body** **involvement *n* (%)**					0.24 ^1^	0.13 ^1^	0.011 ^1^
No	12 (80)	6 (100)	10 (37)	8 (73)			
Yes	3 (20)	0 (0)	17 (63)	3 (27)			
**Cell type**					0.78 ^1^	0.70 ^1^	0.043 ^1^
Spindle	8 (53)	3 (50)	5 (19)	3 (27)			
Mixed	6 (40)	3 (50)	17 (63)	7 (64)			
Epithelioid	1 (7)	0 (0)	5 (19)	1 (9)			
**Extra-ocular extension**					-	0.12 ^1^	0.67 ^1^
*<5 mm*	1 (50)	0 (0)	4 (80)	0 (0)			
*>5 mm*	1 (50)	0 (0)	1 (20)	1 (100)			
**Chr. 8q status** ***n* (%)**					0.16 ^1^	0.64 ^1^	<0.001 ^1^
Normal	10 (66)	2 (33)	4 (15)	1 (9)			
Aberrant	5 (33)	4 (67)	23 (85)	10 (91)			
**Chr. 6 status *n* (%)**					0.24 ^1^	0.80 ^1^	<0.001 ^1^
Normal	3 (20)	0 (0)	23 (85)	9 (82)			
Aberrant	12 (80)	6 (100)	4 (15)	2 (18)			

^1^ Pearson’s chi-squared test. ^2^ Two-tailed *t*-test (without equal variances). ^3^ mitosis/2 mm^2^. Evaluating the group sizes for the different groups in Table 2 with a Fisher’s exact test yielded no significance either (*p*-value = 0.235). This means that the ratio of M3/D3 patients is not significantly different between the type of mutation.

**Table 3:** Mean expression of mRNA expression of HLA Class I antigens and infiltrating immune cells in relation to the *GNA11* or *GNAQ* mutation. *p*-values were calculated using the Wilcoxon-Rank sum test. STD = standard deviation.

mRNA Probe	Illumina	D3 *GNA11* *n* = 9 Mean ± STD	D3 *GNAQ* *n* = 12 Mean ± STD	M3 *GNA11* *n* = 23 Mean ± STD	M3 *GNAQ* *n* = 15 Mean ± STD	M3 *GNA11* versus M3 *GNAQ p*-Value	D3 *GNA11* versus D3 *GNAQ p*-Value	All M3 versus All D3 *p*-Value
**HLA_A**	ILMN 1671054	10.69 ±0.74	10.89 ±0.73	11.85 ±0.84	11.67 ±0.68	0.30	0.34	<0.001
**HLA_A**	ILMN 2203950	13.16 ±0.96	13.38 ±0.64	14.26 ±0.53	14.13 ±0.49	0.42	0.13	<0.001
**HLA_A**	ILMN 2186806	9.87 ±2.08	9.89 ±0.89	11.31 ±1.07	11.13 ±1.22	0.79	0.27	<0.001
**HLA_B**	ILMN 1778401	10.25 ±2.24	10.18 ±1.09	11.98 ±1.40	12.05 ±1.10	0.95	0.21	<0.001
**HLA_C**	ILMN 2150787	6.57 ±0.74	6.22 ±0.41	6.52 ±0.64	6.53 ±0.75	0.46	0.21	0.38
**HLA_C**	ILMN 1721113	7.63 ±1.18	7.46 ±0.53	8.20 ±1.21	8.35 ±1.13	0.70	0.80	0.013
**HLA_E**	ILMN 1765258	11.10 ±1.52	10.84 ±0.59	11.83 ±1.11	11.73 ±0.85	0.74	0.75	<0.001
**HLA_F**	ILMN 1762861	7.65 ±1.18	7.65 ±0.70	8.64 ±1.17	8.46 ±0.97	0.68	0.59	<0.001
**HLA_G**	ILMN 1656670	8.25 ±1.48	8.08 ±0.64	9.05 ±0.76	8.88 ±0.70	0.39	0.70	<0.001
**CD68**	ILMN 1714861	10.36 ±1.30	10.37 ±0.86	11.07 ±0.75	11.01 ±0.82	0.95	0.70	0.008
**CD3D**	ILMN 2261416	7.07 ±1.70	6.66 ±0.40	7.28 ±1.12	7.28 ±1.16	0.95	0.92	0.03
**CD3D**	ILMN 2325837	7.14 ±1.71	6.57 ±0.43	7.37 ±1.23	7.31 ±1.16	0.98	0.46	0.022
**CD3E**	ILMN 1739794	6.56 ±0.50	6.38 ±0.07	6.51 ±0.28	6.57 ±0.31	0.55	0.50	0.19
**CD3G**	ILMN 1717197	6.68 ±0.45	6.53 ±0.11	6.63 ±0.28	6.57 ±0.29	0.39	0.64	0.77
**CD8A**	ILMN 1768482	7.30 ±2.06	6.78 ±0.45	7.45 ±1.23	7.54 ±1.29	0.86	0.86	0.06
**CD8A**	ILMN 1760374	6.58 ±0.58	6.36 ±0.10	6.47 ±0.29	6.54 ±0.45	0.63	0.27	0.25
**CD8A**	ILMN 2353732	7.22 ±2.11	6.65 ±0.43	7.42 ±1.25	7.46 ±1.31	0.98	0.80	0.022
**CD4**	ILMN 1727284	6.65 ±0.49	6.54 ±0.29	6.69 ±0.24	6.73 ±0.36	0.98	0.86	0.044

**Table 4:** Mean expression of mRNA expression of HLA Class I antigens and infiltrating immune cells in relation to Q209L or Q209P mutation on either *GNAQ* or *GNA11*. *p*-values were calculated using the Wilcoxon-Rank sum test. STD = standard deviation.

mRNA Probe	Illumina	D3 Q209L *n* = 15 Mean ± STD	D3 Q209P *n* = 6 Mean ± STD	M3 Q209L *n* = 27 Mean ± STD	M3 Q209P *n* = 11 Mean ± STD	M3 Q209L versus M3 Q209P *p*-Value	D3 Q209L versus D3 Q209P *p*-Value	All M3 versus All D3 *p*-Value
**HLA_A**	ILMN 1671054	10.88 ±0.73	10.63 ±0.72	11.85 ±0.79	11.60 ±0.74	0.30	0.79	<0.001
**HLA_A**	ILMN 2203950	13.35 ±0.80	13.12 ±0.75	14.23 ±0.51	14.16 ±0.55	0.87	0.91	<0.001
**HLA_A**	ILMN 2186806	10.08 ±1.63	9.39 ±0.93	11.20 ±1.10	11.33 ±1.22	0.70	0.37	<0.001
**HLA_B**	ILMN 1778401	10.34 ±1.78	9.89 ±1.26	11.99 ±1.34	12.05 ±1.17	1.00	0.97	<0.001
**HLA_C**	ILMN 2150787	6.34 ±0.66	6.44 ±0.37	6.44 ±0.63	6.74 ±0.77	0.46	0.51	0.38
**HLA_C**	ILMN 1721113	7.57 ±0.95	7.43 ±0.58	8.16 ±1.19	8.50 ±1.12	0.42	0.97	0.013
**HLA_E**	ILMN 1765258	11.01 ±1.21	10.81 ±0.61	11.82 ±1.05	11.71 ±0.93	0.63	1.00	<0.001
**HLA_F**	ILMN 1762861	7.72 ±1.00	7.49 ±0.69	8.58 ±1.13	8.54 ±1.02	1.00	0.67	<0.001
**HLA_G**	ILMN 1656670	8.32 ±1.16	7.73 ±0.61	9.00 ±0.76	8.96 ±0.69	0.85	0.15	<0.001
**CD68**	ILMN 1714861	10.39 ±1.16	10.30 ±0.75	11.07 ±0.74	10.99 ±0.87	0.80	0.85	0.008
**CD3D**	ILMN 2261416	6.92 ±1.32	6.61 ±0.45	7.23 ±1.06	7.41 ±1.29	0.85	0.51	0.033
**CD3D**	ILMN 2325837	6.94 ±1.39	6.49 ±0.47	7.31 ±1.17	7.44 ±1.29	0.75	0.23	0.016
**CD3E**	ILMN 1739794	6.49 ±0.39	6.37 ±0.09	6.51 ±0.26	6.60 ±0.35	0.56	0.41	0.19
**CD3G**	ILMN 1717197	6.60 ±0.36	6.58 ±0.13	6.61 ±0.27	6.60 ±0.33	0.70	0.51	0.77
**CD8A**	ILMN 1768482	7.10 ±1.59	6.75 ±0.58	7.39 ±1.17	7.71 ±1.43	0.56	0.67	0.061
**CD8A**	ILMN 1760374	6.50 ±0.46	6.35 ±0.09	6.47 ±0.27	6.57 ±0.52	0.80	0.41	0.25
**CD8A**	ILMN 2353732	7.00 ±1.63	6.62 ±0.55	7.35 ±1.19	7.64 ±1.44	0.52	0.46	0.019
**CD4**	ILMN 1727284	6.62 ±0.41	6.51 ±0.21	6.70 ±0.25	6.72 ±0.40	0.90	0.61	0.036

**Table 5:** Probes used in the analysis.

Gene	Probe
**HLA-A**	ILMN_1671054
**HLA-A**	ILMN_2203950
**HLA-A**	ILMN_2186806
**HLA-B**	ILMN_1778401
**HLA-C**	ILMN_2150787
**HLA-C**	ILMN_1721113
**HLA-E**	ILMN_1765258
**HLA-F**	ILMN_1762861
**HLA-G**	ILMN_1656670
**CD68**	ILMN 1714861
**CD3D**	ILMN 2261416
**CD3D**	ILMN 2325837
**CD3E**	ILMN 1739794
**CD3G**	ILMN 1717197
**CD8A**	ILMN 1768482
**CD8A**	ILMN 1760374
**CD8A**	ILMN 2353732
**CD4**	ILMN 1727284
